# Respiratory motion navigated Look-Locker imaging for small animal myocardial T1mapping

**DOI:** 10.1186/1532-429X-17-S1-P20

**Published:** 2015-02-03

**Authors:** Pan-ki Kim, Joonsung Lee, Byoung Wook Choi

**Affiliations:** 1 Yonsei University, Seoul, Korea, the Republic of

## Background

The quantification of T1 relaxation time has become an important indicator for diffuse cardiomyopathies. In small animal studies, such as mouse and rat, fast heart beats and respiratory rates are major obstacles to use clinical T1 mapping methods. For small animal T1 mapping, SALLI(Messroghli DR et al., 2011) and mCINE-IR(Smit H et al., 2014) had been reported using the Look-Locker scheme. Since the Look-Locker recovery evolution has to be consistently maintained, the respiratory gating is particularly challenging. In general, the multiple averages were applied to avoid motion artifacts. In this study, respiratory motion navigated Look-Locker imaging (NALLI) was proposed to overcome respiratory motion artifacts for small animal myocardial T1 mapping. To evaluate feasibility, the proposed method was performed for phantoms and a normal mouse.

## Methods

**Figure 1:**
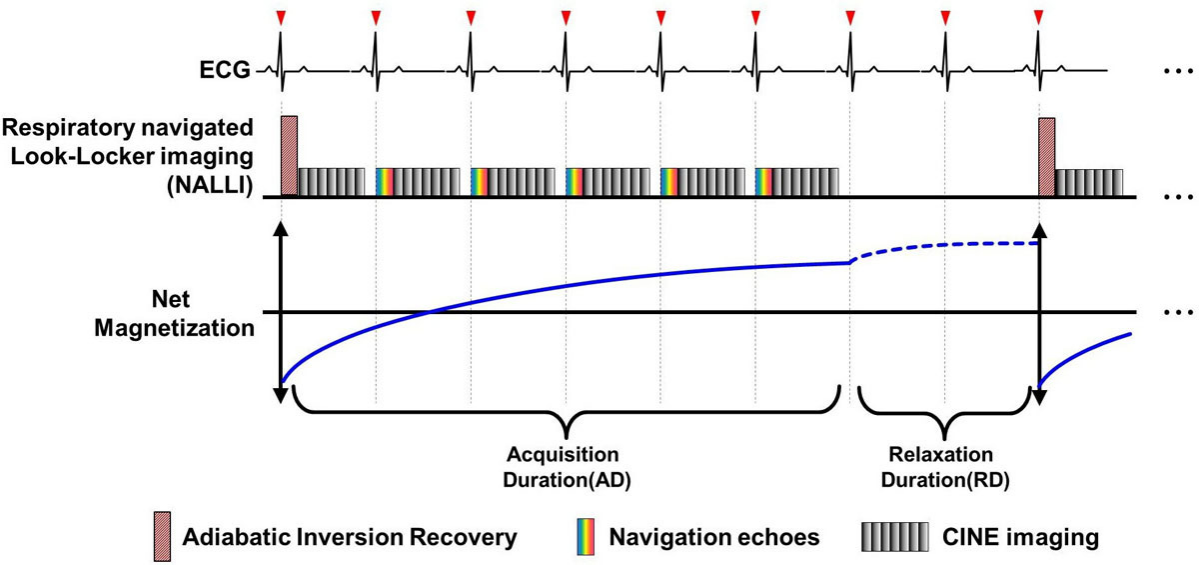
The pulse sequence diagram for describing the respiratory navigated Look-Locker imaging (NALLI). T1weighted images were acquired during the acquisition duration(AD) along prospectively ECG gated CINE imaging fashion. The relaxation duration(RD) is a quiescent time to the fully recovery of the net magnetization toward the equilibrium magnetization. The beginning of AD, The Inversion recovery pulse drives to invert the net magnetization, and then perform the cine imaging with multislice. The navigation scans were performed by measuring the center of k-space to detecting motion-corrupted cardiac cycles. Subsequent, the cine imaging is repeated until the end of AD.

All MR studies were carried out on a 9.4 Tesla MRI (Bruker, Germany). The use of a mouse (C57BL/6) was approved by Institutional Animal Care and Use Committee (IACUC). NALLI was employed the Look-Locker scheme with navigator at the beginning of cardiac cycles as illustrated in Fig. 1. The navigators measured MR signals at the center of k-space to detect motion-corrupted cardiac cycles. The echoes at the motion-corrupted cardiac cycle were replaced by the average of echoes at adjacent cardiac cycles. Look-Locker correction were applied at each pixel. In phantom studies, the NALLI was tested using eight phantoms doped with different amounts of a Gd-DOTA (DOTAREM, Guerbet). The phantoms were attached to an airbag, which inflated and deflated alternately to mimic respiratory motion. Imaging parameters were as follows: acquisition duration = 20 cardiac cycles, relaxation duration=2000 ms, 13 cardiac phases, 3 slices, TR/TE=12/1.25 ms, FA = 10°, FOV=3x2 cm, matrix = 128x86, 0.23x0.23 pixel size, slice thickness = 1.5mm, 300 BPM and 42 RPM. A normal mouse myocardium was scanned under anesthesia with isoflurane. Imaging parameters were same as phantom studies except slice thickness = 1.0mm. The NALLI and conventional Look-Locker imaging (CLL) with multiple averaging were compared to evaluate the motion resistance by measuring T1 value and heterogeneity, defined as coefficient of variations of T1 value, on region of interests.

## Results

**Figure 2:**
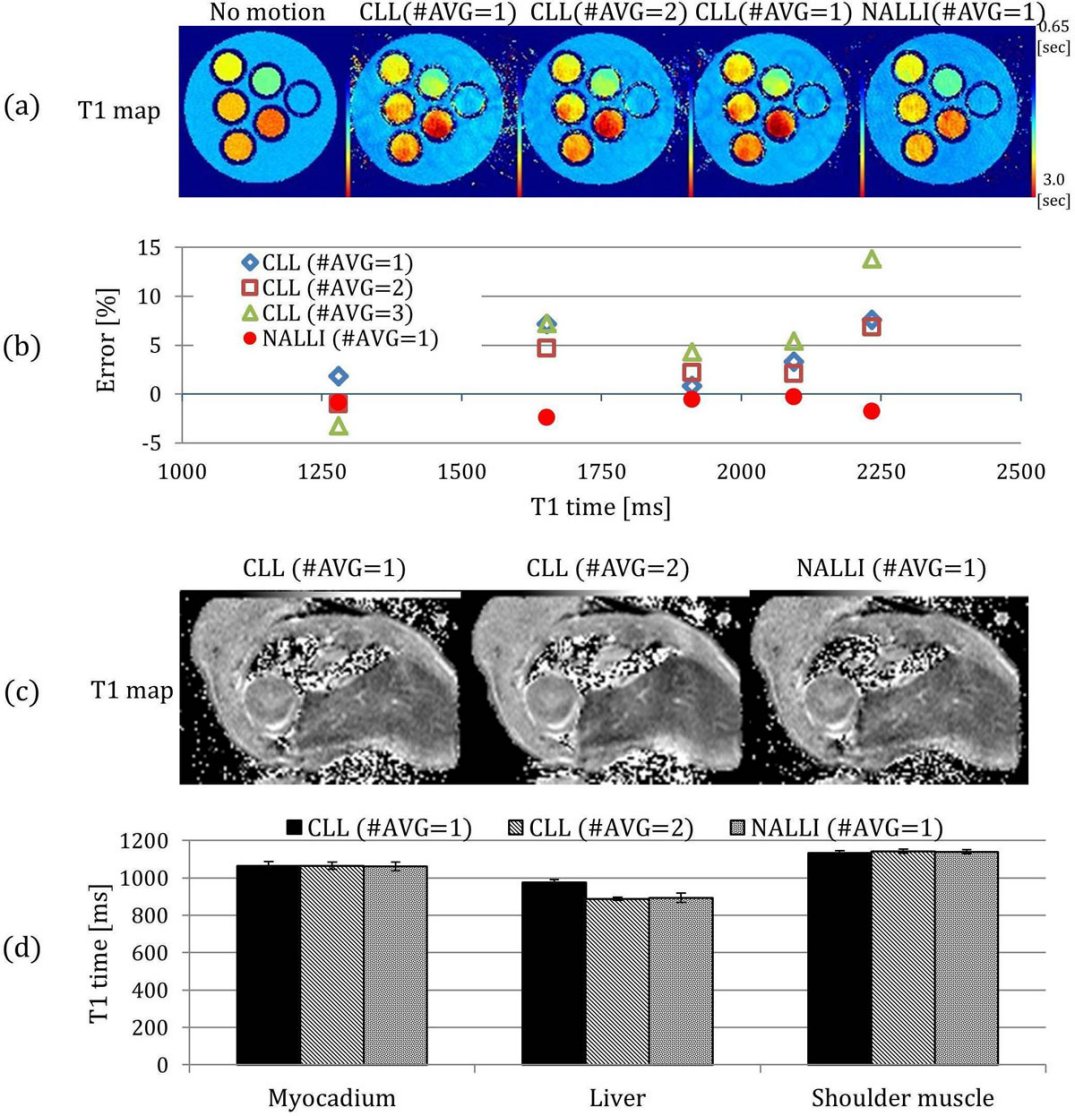
T1 maps and its measurements on phantoms and a mouse. (a) T1 maps using the NALLI and conventional Look-Locker (CLL) with multiple averages on stationary and moving phantom, (b) graphs show accuracy between the NALLI and CLL with difference average on moving phantom. Accuracy is expressed as percentage T1 error from stationary phantom, (c) T1 maps and (d) T1 value on invivo mouse. The T1 map of NALLI and CLL with average 2 have similar T1 values on all ROIs. However, the T1 value of CLL with average 1 has overestimate than the others on liver.

In Fig. 2(a, b), T1 accuracies on moving phantoms were shown as percentage error from stationary phantoms. The NALLI as a mean error of -1.34% has higher accuracy than CLL with multiple averaging on moving phantom. The heterogeneities of NALLI and CLL with average 3 were 3.1% and 3.7%, respectively. The NALLI has been shown as effective as using 3 averages within 1 average time. In fig. 2(c, d), the NALLI and CLL with average 2 produced similar T1 values on all ROI, but T1 maps using CLL with average 1 were overestimated over liver.

## Conclusions

The proposed NALLI method can be provided the higher motion robustness and accuracy T1 map for small animal myocardium without additional measurement required

## Funding

N/A.

